# Perceived stress is associated with impaired artery elasticity: An observational study from the Vara- Skövde cohort

**DOI:** 10.1371/journal.pone.0336298

**Published:** 2025-11-13

**Authors:** Gábor Szaló, Maria C.M. Eriksson, Margareta Hellgren, Kristin Ottarsdottir, Ying Li, Yun Chen, Karin Rådholm, Lyndia C. Brumback, Matthew Allison, Ulf Lindblad, Bledar Daka

**Affiliations:** 1 Family Medicine, School of Public Health and Community Medicine, Institute of Medicine, Sahlgrenska Academy, University of Gothenburg, Gothenburg, Sweden; 2 Biostatistics, School of Public Health and Community Medicine, Institute of Medicine, Sahlgrenska Academy, University of Gothenburg, Gothenburg, Sweden; 3 School of Public Health and Community Medicine, Institute of Medicine, Sahlgrenska Academy, University of Gothenburg, Gothenburg, Sweden; 4 Primary Health Care Centre Kärna, and Department of Health, Medicine and Caring Sciences, Faculty of Medicine and Health Sciences, Linköping University, Linköping, Sweden; 5 Collaborative Health Studies Coordinating Center, University of Washington, Seattle, Washington, United States of America; 6 Division of Preventive Medicine, Department of Family Medicine, University of California San Diego, San Diego, California, United States of America; City of Hope National Medical Center, UNITED STATES OF AMERICA

## Abstract

**Background:**

This study aims to determine the association between perceived mental stress and arterial elasticity. Additionally, we will investigate potential effect modifications of sex and antihypertensive medication on this association.

**Methods:**

Cross-sectional observational study based on the Vara- Skövde Cohort. After excluding individuals with missing information on perceived stress or vascular elasticity, 1015 individuals remained. Perceived stress was evaluated with the Perceived Stress Scale-10 (PSS-10). Small Artery Elasticity Index (SAEI or C2) was estimated from a Windkessel model obtained by applanation tonometry over the arteria radialis. Impaired artery elasticity was defined as the lowest sex-specific quartile of C2. The associations between perceived stress and artery elasticity indices were investigated using linear and logistic regressions with adjustments for possible confounding in different models. Due to significant interactions tests we stratified for men and women and for individuals with and without antihypertensive medication.

**Results:**

The mean age of study participants was 57 years and women reported significantly higher stress levels on PSS-10 than men [Women: 13.6 ± 5.6; Men: 12.4 ± 5.3; p < 0.01]. Among those 803 individuals who did not take antihypertensive medication, there was a significant association in linear regression between increase in PSS-10 and decrease in C2 (B: −0.2, 95% CI: −0.4- −0.02; p = 0.03) that was lost after adjustment for physical activity (B: −0.16, 95% CI: −0.35–0.03; p = 0.1). In logistic regression analyzes, each increment with 1-SD in PSS-10 was associated with 25% higher odds of having impaired artery elasticity in all models (OR=1.25, 95% CI: 1.01–1.55; p = 0.04).

**Conclusions:**

Among middle-aged adults without antihypertensive medication, higher perceived stress was associated with impaired arterial elasticity.

## Introduction

Mental stress and illness are important risk factors for atherosclerosis, cardiovascular (CV) morbidity and mortality [1–3]. Mental stress affects vascular health through increased risk for unhealthy behavior (e.g., physical inactivity eating habits, smoking, excessive alcohol consumption) and biological mechanisms (e.g., inflammatory responses, overactivation of the sympathetic nervous system and the hypothalamic-pituitary-adrenal axis, including hypercortisolism and endothelial dysfunction) [1–5].

The Perceived Stress Scale (PSS) is a frequently used instrument to measure perceived stress that focuses on the subjective appraisal of life stress, rather than on objective measures of the impact of life events. It evaluates whether an individual has perceived life as unpredictable, uncontrollable and overloading [6]. The 10-item version of the PSS is recommended in clinics and research due to its superiority in psychometric properties [7].

Impairment of vascular elasticity occurs before and in parallel with atherosclerotic changes [8,9]. Briefly, a deterioration process characterized by a reduction in the quantity of elastin fibers, an increase in collagen, destroying muscle fibers, and forming calcium deposits in the tunica media occurs with aging and accelerates due to atherosclerosis. This leads to changes in the pulse waveform, which occurs before developing elevated pulse pressure and systolic hypertension [8,9]. Notably, impaired vascular elasticity predicts cardiovascular events [10–12] potentially beyond conventional risk factors, and it is considered a valuable marker for cardiovascular risk prediction [10,11,13–16].

Although previous studies have investigated the association between perceived stress and the development of hypertension [17–19], relatively few studies have investigated how vascular elasticity is affected by perceived stress [5,20–25], and all of those studies include relatively small groups [20–23,25] or children and adolescents [24]. As such, the association between mental stress and vascular elasticity in a larger middle-aged population has not been fully investigated, nor have sex differences been examined. Therefore, this study aims to examine the association between perceived mental stress and arterial elasticity, as measured by diastolic pulse wave analysis, in middle-aged adults. Additionally, we will investigate potential effect modifications of sex and antihypertensive medication on this association.

## Methods

### Study population, design and measurements

**Fig 1:**
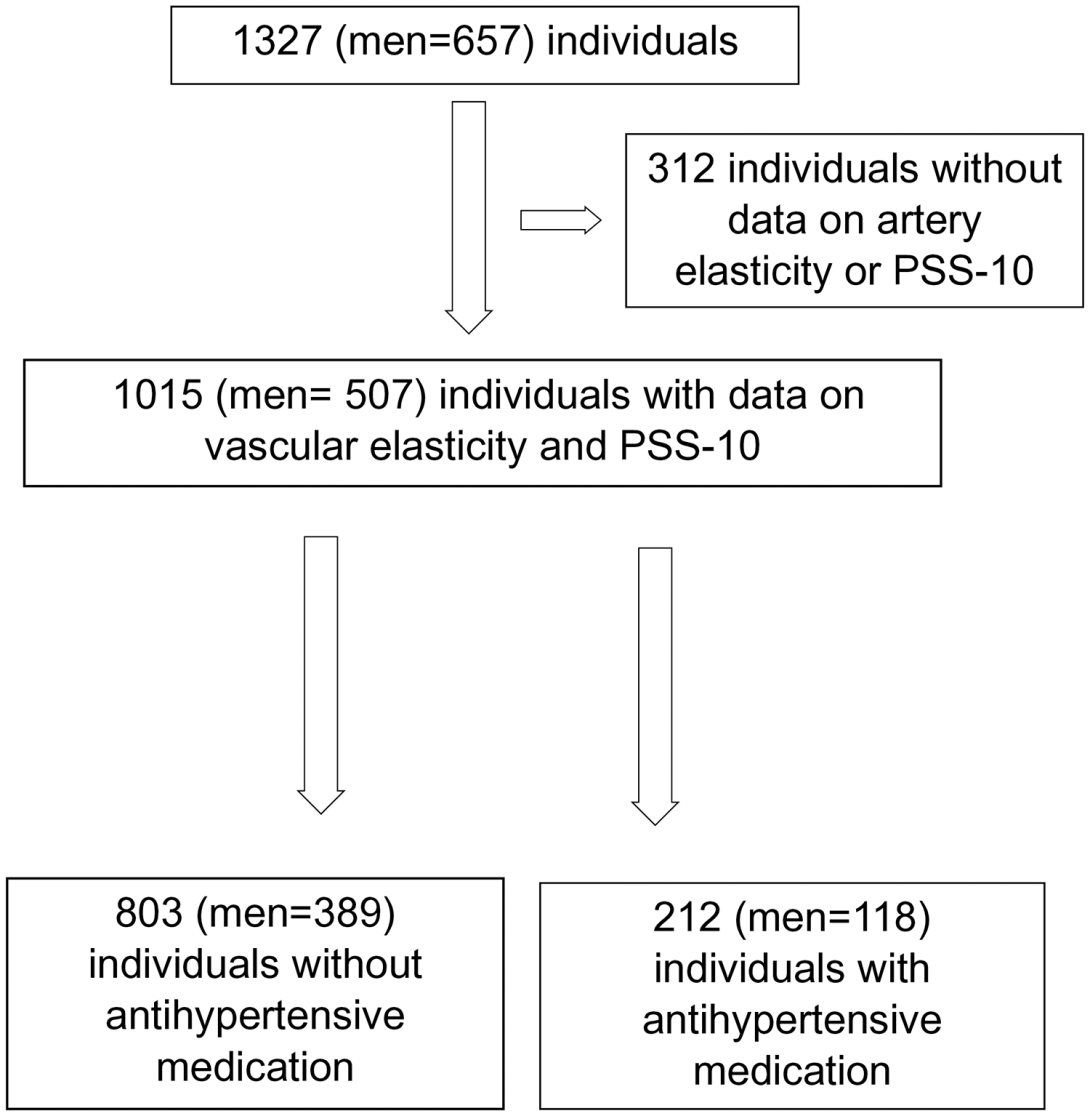
Flowchart of the study population.

This cross-sectional study is based in Vara and Skövde municipalities in south-western Sweden, designed to detect early signs and risk factors for cardiovascular disease (CVD) [26]. We used information collected on the second visit, conducted between September 11, 2012, and August 1, 2014, including 1327 individuals (657 men), who agreed to participate in the second visit using the same protocol as at baseline [27]. After we excluded individuals with missing information on vascular elasticity (N = 241) and perceived stress scale 10 (N = 71), we included 1015 (507 men) individuals in this study (Fig 1).

*Perceived stress* was evaluated with the PSS-10 (Perceived Stress Scale) [28]. The PSS-10 contains ten questions with a 5-point Likert scale that ranges from 0 (never) to 4 (very often), thus the total score of PSS-10 ranged from 0 to 40. The PSS-10 scale focuses on the perceived stress in the last month. The scale used in this study has been previously developed and validated across various cultural contexts, including Sweden [28,29]. The PSS-10 is not a diagnostic instrument and the developer has not published any score cut-offs useful for clinical purposes [28]. The validation study of the Swedish version of PSS-10 [29] showed that 10% of individuals scored 22 points or higher in the entire study population, 26 points for women and 20 points for men. Based on these findings, stress levels were categorized as follows: low to moderate stress was defined as a PSS-10 score of ≤19 for men and ≤21 for women (including a score of 0), while high stress was defined as a score of ≥20 for men and ≥22 for women.

*Artery elasticity* was estimated using an applanations tonometer (Pulsewave CR-2000, Hypertension Diagnostics Inc.) over the radial artery. The participant’s right wrist was immobilized, and the sensor was positioned at the site of maximal radial artery pulsation. Simultaneously, oscillometric systolic and diastolic blood pressure measurements were obtained from the left arm. We performed repeated measurement series 3 times in a row and used a calculated mean value of those three measurements. Indices for the arterial elasticity were obtained based on Windkessel theory [30]. We focused on the Small Artery Elasticity Index (SAEI or C2); a lower value of C2 has been associated with a higher risk of the development of hypertension and cardiovascular disease [12,31,32]. In our previous study [12], individuals in the lowest quartile of C2 were significantly more likely to have incident CVD than the other quartiles of C2. Thus, we defined impaired artery elasticity levels as C2 at the lowest sex-specific quartile. All examinations were performed in the morning.

Information on medical history and lifestyle factors, such as alcohol consumption, smoking, and physical activity, were collected through validated questionnaires. Current smoking status was categorized as a binary variable (yes/no). Leisure-time physical activity (LTPA) was assessed using a self-reported four-level scale, corresponding to the Saltin-Grimby Physical Activity Level Scale (SGPALS), which categorizes participants based on the type and intensity of their regular leisure-time activities [33,34]. Participants were asked to report their activity over the past two weeks. Based on their responses, they were classified as inactive (engaging in light activities such as walking, cycling, or gardening for less than four hours per week), moderately active (light activities for more than four hours per week), strenuous (more intense activities such as jogging, swimming, or tennis for more than two hours per week), or highly strenuous (regular participation in competitive sports on a weekly basis). Alcohol consumption was assessed by asking individuals how many days in the past month they had consumed alcohol. This number was then multiplied by their self-reported daily alcohol intake to calculate the weekly consumption, expressed in grams per week [35]. Information on current medications was collected through an interview with a trained nurse and included detailed information on the name of the medication, indication, dosage (expressed as dose per medication), administration period, and dose per administration occasion. Antihypertensive medication was defined as the prescription of at least one of the following drug classes: Angiotensin-Converting Enzyme Inhibitors, Angiotensin II Receptor Blockers, Alpha- Blockers, Beta-Blockers, Calcium Channel Blockers, Thiazide Diuretics, Aldosterone Antagonists or Loop Diuretics. Based on this information we identified 803 individuals (389 men, 48%) without any antihypertensive medication and 212 individuals (118 men, 56%) with antihypertensive medication (Fig 1).

Body height and weight were recorded in light clothing and without shoes and were rounded to the nearest 1 cm and 0.1 kg. Waist-to-hip ratio (WHR) was calculated by measurements taken between the lowest rib margin and the iliac crest, and at the widest point between the waist and thighs, respectively. Blood pressure was taken in the right arm after a five-minute rest in a lying position and the values were read at the nearest 2 mmHg. Hypertension was defined according to Blood Pressure Management guidelines (JNC-7) [36]. All individuals underwent standard oral glucose tolerance test (OGTT). Participants fasted for 12 hours prior to the test. After fasting, they consumed 75 grams of glucose dissolved in water. Venous blood samples were collected at fasting and at 120 minutes after glucose intake. Diabetes was diagnosed based on the 1999 WHO criteria [37]. Assays for LDL-cholesterol, triglycerides and C-reactive protein (CRP) were performed.

### Statistics

Descriptive statistics were used to present the characteristics of the study population. Linear regression analyses were used to investigate the association between PSS-10 and C2 when both were used as continuous variables. PSS- 10 variable was standardized, and the effect sizes were estimated based on changes in one SD of PSS-10. Logistic regression models were used to investigate the odds of having impaired artery elasticity. General linear models were used to compare vascular elasticity in individuals with high and low-to-moderate levels of stress. We adjusted for possible confounding using a stepwise approach, with each adjustment step based on theoretical models guiding the selection and inclusion of relevant variables. In model 1, we adjusted for age, sex, systolic blood pressure and heart rate. Model 2 included variables in model 1 adding also waist-hip ratio, LDL-cholesterol, and levels of C-reactive protein, smoking, alcohol consumption, presence of statin medication. Finally, model 3, we included all variables in model 2 adding information on physical activity.

To investigate whether the association between perceived stress and vascular elasticity differed between individuals with and without blood pressure-lowering medication, we conducted an interaction test. The interaction term suggested a potential effect modification by medication status (p = 0.057); therefore, we performed stratified analyses. Interaction tests to investigate differences in the associations between men and women were also computed. Investigating the differences in the levels of arterial elasticity between individuals with high and low-to-moderate levels of stress we found that the p-value of interaction for sex and C2 was p = 0.151. All analyses were computed in IBM SPSS 29.

Artificial intelligence tools assisted with language editing of the manuscript.

### Ethics approval and consent to participate

All participants were informed on the study procedures and goals. The study collected written informed consent from all the participants. We confirm that all methods were performed in accordance with relevant guidelines and regulations. This study was approved by the Ethics Committee at the University of Gothenburg, Sweden (DNR: 036−12).

## Results

The mean age in this study population was 57 years. Similar number of men and women participated in this study (Men 507/ Women 508). Men had higher blood pressure values, WHR, fasting glucose, fasting triglycerides, LDL- cholesterol and levels of alcohol consumption than women. We observed significant sex differences in C2 [Mean (SD) for men: 7.32 ml/mmHg × 100 (3.38); for women: 5.91 ml/mmHg × 100 (3.07), p < 0.01]. Women reported significantly higher stress levels on PSS-10 than men [Mean (SD) Women: 13.6 (5.6); Men: 12.4 (5.3); p < 0.01] (S1 Table).

Characteristics of the population with and without antihypertensive medication are presented in Table 1. Compared to those not receiving such treatment (N = 803, 48%), individuals taking antihypertensive medication (N = 212, 20.8%) were significantly older and had significantly lower arterial elasticity and significantly higher systolic blood pressure, BMI, WHR, fasting glucose, HOMA-IR, and triglycerides, inflammatory markers, and prevalence of diabetes. Furthermore, significantly larger proportion reported a sedentary lifestyle. LDL levels were lower in the group receiving antihypertensive treatment; however, a significantly greater proportion of this group was also receiving lipid-lowering medication (Table 1).

**Table 1:** Characteristics of study population.

	All n = 1015	No anti-HT n = 803	Anti-HT n = 212	p-value
Men, n (%)	507 (49.9)	389 (48.4)	118 (55.7)	0.062
Age^a^, years	57.2 (11.2)	54.7 (10.1)	66.5 (10.2)	<0.001
SBP^a^, mmHg	126 (15)	124 (15)	133 (14)	<0.001
DBP^a^, mmHg	77 (10)	77 (10)	77 (10)	0.551
Pulse^a^, bpm	64 (9)	64 (9)	64 (9)	0.787
BMI^a^ kg/m²	27.3 (4.5)	26.7 (4.1)	29.4 (5.2)	<0.001
WHR^a^	0.92 (0.09)	0.91 (0.08)	0.95 (0.08)	<0.001
Fasting glucose^a^, mmol/l	5.6 (1)	5.5 (0.8)	6.1 (1.5)	<0.001
HOMA- IR median (IQR)	1.92 (1.32-2.98)	1.77 (1.26- 2.6)	3.01 (1.87- 4.23)	<0.001
Median of Triglyceride, mmol/l (IQR)	1.06 (0.79-1.41)	1 (0.77- 1.35)	1.22 (0.95- 1.68)	<0.001
LDL cholesterol^a^, mmol/l	3.5 (0.9)	3.6 (0.9)	3.2 (1)	<0.001
Median of CRP, mg/l (IQR)	1.4 (0.7–2.6)	1.2 (0.7- 2.4)	1.8 (1.1- 3.7)	<0.001
Medication with antihyperlipidemics, n (%)	126 (12.4)	27 (3.4)	99 (46.7)	<0.001
Total hypertension, n (%)	264 (26.1)	59 (7.3)	205 (96.7)	<0.001
Total DM, n (%)	94 (9.3)	32 (4)	62 (29.2)	<0.001
Current smoker, n (%)	96 (9.5)	83 (10.3)	13 (6.1)	0.073
Non-drinker, n (%)	185 (18.2)	131 (16.3)	54 (25.5)	0.256
Low-level of physical activity (LTPA: 1 and 2), n (%)	610 (60.1)	467 (58.2)	143 (67.5)	0.008
European descent n (%)	990 (97.5)	782 (97.4)	208 (98.1)	0.543
PSS-10^a^	12.9 (5.5)	13 (5.6)	12.8 (5)	0.694
C2^a^, ml/mmHg × 100	6.61 (3.3)	7.03 (3.3)	5.01 (2.76)	<0.001

^a^: data are presented as means (SD). No anti-HT: individuals with no antihypertensive medication; Anti-HT: individuals on antihypertensive medication; p-value: p values from the tests to the differences between individulals with and without antihypertensive medication, SBP: Systolic Blood Pressure, DBP: Diastolic Blood Pressure, BMI: Body Mass Index, HOMA-IR: Homeostatic Model Assessment for Insulin Resistance, LDL: Low-Density Lipoprotein, DM: Diabetes Mellitus, C2: Small Artery Elasticity, IQR: interquartile range, LTPA: Leisure Time Physical Activity, European descent: individuals who were either born in Europe or had both parents born in Europe.

### Individuals without antihypertensive medication (N=803)

There was a significant linear association between an increase in PSS-10 scores and a decrease in levels of C2 in model 2 (B: −0.2–95% CI: −0.39- −0.01; p = 0.03). In the fully adjusted model, including adjustments for physical activity, the significance in the association was lost (B: −0.16, 95% CI: −0.35–0.03; p = 0.1) (Table 2). Sex-specific analyses were not computed as the p for interaction was 0.88.

**Table 2:** The associations between levels of perceived stress scale-10 and small artery elasticity index (C2) in the Vara-Skövde cohort, in individuals without antihypertensive medication (N = 803).

B	Confidence interval (95% CI)	p-value
Model 1: Adjusted for age, sex, heart rate and systolic blood pressure		
**−0.2**	**−0.38 - −0.02**	**0.03**
Model 2: Adjusted as above + waist-hip -ratio, LDL cholesterol and CRP, alcohol consumption, smoking and statin medication		
**−0.2**	**−0.39 - −0.01**	**0.03**
Model 3: Adjusted as above + physical activity		
**−0.16**	**−0.35 - 0.03**	**0.1**

Linear regression analyses were conducted to examine the associations between the levels of the Perceived Stress Scale (PSS-10) and the Small Artery Elasticity Index (C2) after excluding participants with antihypertensive medication and with missing values. B = the unstandardized coefficient. CRP: C- reactive protein. LDL: low-density lipoprotein.

In logistic regression analyses, there was a significant association between increase in perceived stress and the odds of impaired artery elasticity in all models. Specifically, for each 1-SD increase PSS-10 there was 25% higher odds of having impaired artery elasticity (Model 3: OR=1.25, 95% CI: 1.01–1.55, p = 0.04) (Table 3).

**Table 3:** Associations between perceived stress scale 10 and impaired arterial elasticity in the Vara-Skövde cohort, in individuals without antihypertensive medication (N = 803).

OR	Confidence interval (95% CI)	p-value
Model 1: Adjusted for age, sex, heart rate and systolic blood pressure		
**1.2**	**0.99 - 1.45**	**0.07**
Model 2: Adjusted as above + waist-hip -ratio, LDL cholesterol and CRP		
**1.29**	**1.05 - 1.59**	**0.02**
Model 3: Adjusted as above + alcohol consumption, smoking and physical activity		
**1.25**	**1.01- 1.55**	**0.04**

Logistic regression analyses were used in the cross-sectional examination between 2012 and 2014 after excluding individuals with missing values and antihypertensive medication. Effect sizes are based on change with 1 standard deviation in PSS-10. Impaired artery elasticity is defined as C2 at the lowest sex-specific quartile. Number of the individuals in the lowest quartile of C2: 200: OR: Odds ratio, CRP: C- reactive protein.

**Fig 2:**
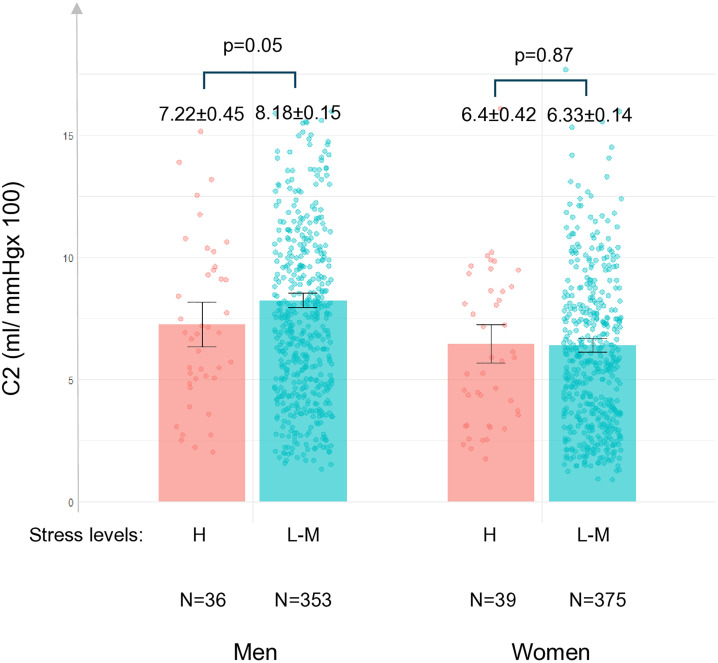
Levels of small artery elasticity index (C2) in individuals with low to moderate and high perceived stress levels. C2 values are reported as mean ± standard error (SE). Analyses are adjusted for age, sex, systolic blood pressure, heart rate, waist-hip ratio, LDL-cholesterol, CRP, smoking, alcohol consumption, presence of statin medication, and leisure time physical activity. Low to moderate (L-M) stress: PSS-10 ≤ 19 for men, ≤ 21 for women. High (H) stress: PSS-10 ≥ 20 for men, ≥ 22 for women. Analysis includes individuals without antihypertensive medication and after excluding missing values (N = 803).

Moreover, men with a high level of perceived stress had lower C2 than men with low to moderate levels of perceived stress after adjustments in model 2 (C2 in a low to moderate level of perceived stress: 8.16 ml/mmHg × 100, 95% CI: 7.87–8.46, C2 in a high level of perceived stress: 7.15 ml/mmHg × 100, 95% CI: 6.27–8.03, difference in means = 1.01 ml/mmHg × 100, 95% CI: 0.09–1.94, p = 0.03). However, when physical activity was included in the model, the association became statistically insignificant (C2 in a low to moderate level of perceived stress: 8.18 ml/mmHg × 100, 95% CI: 7.89–8.48, C2 in a high level of perceived stress: 7.25 ml/mmHg × 100, 95% CI: 6.37–8.14, difference in means = 0.93 ml/mmHg × 100, 95% CI:-0.07–1.87, p = 0.05). These differences were not significant in women in any model (difference in means = 0.07 ml/mmHg × 100, 95% CI: −0.95–0.8, p = 0.87) (Fig 2).

### Individuals with antihypertensive medication (N=212)

There was a significant linear association between an increase in PSS-10 scores and an increase in levels of C2 in the full model (B = 0.42 95% CI: 0.07–0.76; p = 0.02). There was no significant association between PSS-10 scores and the risk of impaired vascular elasticity across all models for participants without antihypertensive medication (Model 3: OR=0.85, 95% CI: 0.56–1.28, p = 0.43). Men with a high level of perceived stress had significantly higher C2 than men with low to moderate levels of perceived stress in model 3 (C2 in a low to moderate level of perceived stress: 5.22 ml/mmHg × 100, 95% CI: 4.74–5.7, C2 in a high level of perceived stress: 8.24 ml/mmHg × 100, 95% CI: 6.73–9.74, difference in means = 3.01 ml/mmHg × 100, 95% CI: 1.41–4.62, p= < 0.01). These differences were not significant in women in any model (difference in means = 0.37 ml/mmHg × 100, 95% CI: −1.34 2.08, p = 0.67).

A non-participation analysis was performed comparing individuals with and without information on vascular elasticity (N = 241) and PSS-10 (N = 71). No difference in the distribution of gender or mean systolic blood pressure levels was observed between participants and non-participants (p = 0.586 and p = 0.832, respectively). However, non-participants were significantly older (participants vs. non-participants: difference in means = 6.7 years, SD ± 1.4; p < 0.01)

## Discussion

### Main findings

Among middle-aged adults without antihypertensive medication, we observed a significant association between increase in levels of perceived stress and impaired artery elasticity in the overall population and similar results were obtained when both variables were analyzed as continuous. However, this association was not consistently observed across all analytic approaches or subgroups; for example, when perceived stress was treated as a binary variable, no significant association was found among women. These findings suggest that the relationship between stress and vascular function may be complex and potentially influenced by sex-specific or methodological factors, such as the choice of stress thresholds.

Interestingly, opposite associations were observed in individuals on antihypertensive treatment where an increase of self-reported stress was associated with increased arterial elasticity. While we cannot rule out the possibility of a type I error, this finding should be interpreted with caution. This counterintuitive finding may partly be attributable to the effects of antihypertensive and lipid-lowering medications [38,39], which are known to have a generally beneficial impact on vascular function. However, individuals with higher stress levels and psychiatric comorbidities are often associated with lower adherence to the medication compared to those without such conditions [40], which could potentially attenuate these positive effects. It is worth noting, though, that middle-aged persons tend to have a better adherence to medication regimens than other age groups [41], which may help to explain the observed association in this age group. Overall, the study found no significant associations between perceived stress and arterial elasticity among those on blood pressure-lowering medication.

To our knowledge, this is the first large population study that investigated the associations between perceived stress and artery elasticity in middle-aged adults. Previous studies have investigated whether acute stress affects artery elasticity. These had a limited number of participants (the highest number of participants in those studies 85 individuals) [20–23] or included children [24].

Our analyses focused on perceived stress which encompasses the perception of lack of control and coping, thus a more complex assessment of stress [28,29]. A previous study [25] examined emotional stress using multiple instruments, including PSS-10 in Korean individuals and found a significant association with arterial stiffness measured in larger arteries via the SphygmoCor system. In contrast, our study assessed perceived stress using the PSS-10 in a larger population, measuring elasticity in smaller resistance arteries. We also accounted for physical activity and stratified by antihypertensive medication use, offering broader generalizability and more comprehensive adjustments. Similar to this study, the Cardiovascular Risk in Young Finns study examined the associations between chronic stress and arterial elasticity and found that men experiencing vital exhaustion have reduced vascular elasticity and increased risk for atherosclerosis [42]. The mean age of about 31 years was younger than in our cohort and we could show that similar results apply also in an older cohort as ours. One possible explanation of our findings includes the long-term mental stress triggers pathological mechanisms such as overactivation in the sympathetic nervous system and the hypothalamic-pituitary-adrenal axis, including hypercortisolism [3,43]. In fact, it has been shown that stress induces disturbances in the inflammatory system and the prothrombic system and increases the risk of endothelial dysfunction and atherosclerosis [3,5,43,44]. All the above factors might contribute to reduced artery elasticity due to perceived stress.

Leisure time physical activity (LTPA) is protective of the deleterious effects of stress [45]. Meanwhile, in a recent publication [46], the high self-reported LTPA was associated with better vascular elasticity. In model 3, adjusting for LTPA attenuated the effect size in the linear association between arterial elasticity and perceived stress. However, the association between impaired arterial elasticity and perceived stress remained significant across all models, confirming the confounding role of LTPA.

This study indicated sex-difference in the association between stress and C2. Among those without antihypertensive medication, high levels of perceived stress were associated with lower artery elasticity in men, but not in women. This sex difference aligns with some previous studies [25,37]. Sex differences in these associations might be traced back to differences in sex hormones, the renin-angiotensin-aldosterone system, oxidative stress and inflammation pathways [47] and differences in coping patterns between men and women [48].

### Strengths and limitations

The strength of our study was the high participation rate. Furthermore, levels and the distribution of PSS-10 and C2 shows also a similarity with previous studies where women had higher PSS-10 values [28,29] and lower C2 than men [12,46,49] confirming the external validity in the measurements. By performing three sequential measurements of C2, we reduced random variability and improved measurement precision. All examinations were performed in the morning to minimize circadian variation in vascular measurements [50].

C2 is derived from the diastolic waveform, systolic and diastolic blood pressure, height, weight, age, and heart rate. Therefore, the observed association between stress and C2 may be partially explained by the association between stress and those other measures. The lack of information in adherence to the medication is a weakness in this study and might have led to a Type I error in this study, therefore further investigations in the associations between vascular elasticity and stress are warranted. Smoking and alcohol habits were assessed through self-report. No data on diet, drug misuse or addiction were collected. This is a cross-sectional observational study, and we cannot exclude the risk of residual confounders or reverse causality. The study cohort consisted almost exclusively of individuals of European descent. This may limit the generalizability of our findings, and future studies should test the proposed theory in more diverse populations.

### Clinical implications

We found that high levels of perceived stress were associated with lower arterial elasticity among middle-aged individuals not taking antihypertensive medication. Perceived stress might increase the risk of developing hypertension and cardiovascular disease. Intervention to reduce stress levels might be important to prevent hypertension and cardiovascular diseases. Interestingly, among individuals taking blood pressure-lowering medication, the study did not observe a consistent negative association between perceived stress and arterial elasticity. One possible explanation is that such medications may exert vascular protective effects, thereby buffering the negative influence of stress on arterial elasticity. Therefore, it is important that individuals who require blood pressure-lowering treatment take their prescribed antihypertensive medications consistently, as doing so can help maintain healthier arteries and reduce the risk of cardiovascular disease.

## Supporting information

S1 Table Characteristics of study population, divided by sex. ^a^: data are presented as means (SD). p-value: p values from the tests to the differences between men and women, SBP: Systolic Blood Pressure, DBP: Diastolic Blood Pressure, BMI: Body Mass Index, HOMA-IR: Homeostatic Model Assessment for Insulin Resistance, LDL: Low-Density Lipoprotein, DM: Diabetes Mellitus, C2: Small Artery Elasticity, IQR: interquartile range, LTPA: Leisure Time Physical Activity.
(DOCX)

## Abbreviations

95% CI: Confidence interval
CRP: C-reactive protein
CVD: cardiovascular disease
C2: Small Artery Elasticity Index
DM: diabetes mellitus
LDL: low-density lipoprotein
OR: Odds Ratio
LTPA: Leisure Time Physical Activity
PSS-10: Perceived Stress Scale- 10
SAEI: Small Artery Elasticity Index
SD: Standard deviation
WHR: waist-hip ratio.
