# Supplementary material for: Perceived stress is associated with impaired artery elasticity: An observational study from the Vara- Skövde cohort
Source: PLoS One. 2025 Nov 13;20(11):e0336298. doi: 10.1371/journal.pone.0336298 (PMC12614591; doi:10.1371/journal.pone.0336298)
Supplement: S1 Table — a: data are presented as means (SD). p-value: p values from the tests to the differences between men and women, SBP: Systolic Blood Pressure, DBP: Diastolic Blood Pressure, BMI: Body Mass Index, HOMA-IR: Homeostatic Model Assessment for Insulin Resistance, LDL: Low-Density Lipoprotein, DM: Diabetes Mellitus, C2: Small Artery Elasticity, IQR: interquartile range, LTPA: Leisure Time Physical Activity. (DOCX) [file pone.0336298.s001.docx]

| **Supplementary table. Characteristics of study population, divided by sex** | | | | | |
| --- | --- | --- | --- | --- | --- |
|  | **All n=1015** | **Men n=507** | | **Women n=508** | **p-value** |
| Age^a^, years | 57.2 (11.2) | | 57.9 (11.7) | 56.4 (10.6) | 0.039 |
| SBP^a^, mmHg | 126 (15) | | 127 (14) | 124 (16) | <0.001 |
| DBP^a^, mmHg | 77 (10) | | 78 (10) | 75 (10) | <0.001 |
| Pulse^a^, bpm | 64 (9) | | 64 (9) | 65 (9) | 0.099 |
| BMI^a^ kg/m² | 27.3 (4.5) | | 27.3 (3.9) | 27.3 (5.1) | 0.974 |
| WHR^a^ | 0.92 (0.09) | | 0.97 (0.06) | 0.86 (0.08) | <0.001 |
| Fasting glucose^a^, mmol/l | 5.6 (1) | | 5.8 (1.1) | 5.4 (0.8) | <0.001 |
| HOMA- IR median (IQR) | 1.92 (1.32-2.98) | | 2.17 (1.47-3.17) | 1.69 (1.19-2.67) | 0.010 |
| Median of Triglyceride, mmol/l (IQR) | 1.06 (0.79-1.41) | | 1.18 (0.88-1.57) | 0.95 (0.74-1.26) | <0.001 |
| LDL cholesterol^a^, mmol/l | 3.5 (0.9) | | 3.5 (1) | 3.4 (0.9) | 0.043 |
| Median of CRP, mg/l (IQR) | 1.4 (0.7–2.6) | | 1.4 (0.8- 2.5) | 1.4 (0.7- 2.8) | 0.720 |
| Medication with antihyperlipidemics, n (%) | 126 (12.4) | | 80 (15.8) | 46 (9.1) | 0.001 |
| Total hypertension, n (%) | 264 (26.1) | | 146 (28.8) | 118 (23.3) | 0.045 |
| Total DM, n (%) | 94 (9.3) | | 58 (11.4) | 36 (7.1) | 0.015 |
| Current smoker, n (%) | 96 (9.5) | | 42 (8.3) | 54 (10.6) | 0.197 |
| Non-drinker, n (%) | 185 (18.2) | | 74 (14.6) | 111 (21.9) | <0.001 |
| Low-level of physical activity (LTPA: 1 and 2), n (%) | 610 (60.1) | | 309 (60.9) | 301 (59.3) | 0.360 |
| PSS-10^a^ | 12.9 (5.5) | | 12.4 (5.3) | 13.6 (5.6) | <0.001 |
| C2^a^, ml/mmHg×100 | 6.61 (3.3) | | 7.32 (3.38) | 5.91 (3.07) | <0.001 |

^a^: data are presented as means (SD). p-value: p values from the tests to the differences between men and women, SBP: Systolic Blood Pressure, DBP: Diastolic Blood Pressure, BMI: Body Mass Index, HOMA-IR: Homeostatic Model Assessment for Insulin Resistance, LDL: Low-Density Lipoprotein, DM: Diabetes Mellitus, C2: Small Artery Elasticity, IQR: interquartile range, LTPA: Leisure Time Physical Activity
